# The role of nanoparticles in plant biochemical, physiological, and molecular responses under drought stress: A review

**DOI:** 10.3389/fpls.2022.976179

**Published:** 2022-11-24

**Authors:** Adnan Rasheed, Huijie Li, Majid M. Tahir, Athar Mahmood, Muhammad Nawaz, Adnan Noor Shah, Muhammad Talha Aslam, Sally Negm, Mahmoud Moustafa, Muhammad Umair Hassan, Ziming Wu

**Affiliations:** ^1^ Key Laboratory of Plant Physiology, Ecology and Genetic Breeding, Ministry of Education/College of Agronomy, Jiangxi Agricultural University, Nanchang, Jiangxi, China; ^2^ College of Humanity and Public Administration, Jiangxi Agricultural University, Nanchang, China; ^3^ Department of Soil and Environmental Sciences, Faculty of Agriculture, University of Poonch, Rawalakot, Pakistan; ^4^ Department of Agronomy, University of Agriculture Faisalabad, Faisalabad, Pakistan; ^5^ Department of Agricultural Engineering, Khwaja Fareed University of Engineering and Information Technology, Rahim Yar Khan, Punjab, Pakistan; ^6^ Life Sciences Department, College of Science and Art, King Khalid University, Mohail, Saudi Arabia; ^7^ Unit of Food Bacteriology, Central Laboratory of Food Hygiene, Ministry of Health, Sharkia, Egypt; ^8^ Department of Biology, College of Science, King Khalid University, Abha, Saudi Arabia; ^9^ Botany and Microbiology Department, Faculty of Science, South Valley University, Qena, Egypt; ^10^ Research Center on Ecological Sciences, Jiangxi Agricultural University, Nanchang, China

**Keywords:** antioxidant, drought stress, genes expression, nanoparticles, photosynthesis

## Abstract

Drought stress (DS) is a serious challenge for sustaining global crop production and food security. Nanoparticles (NPs) have emerged as an excellent tool to enhance crop production under current rapid climate change and increasing drought intensity. DS negatively affects plant growth, physiological and metabolic processes, and disturbs cellular membranes, nutrient and water uptake, photosynthetic apparatus, and antioxidant activities. The application of NPs protects the membranes, maintains water relationship, and enhances nutrient and water uptake, leading to an appreciable increase in plant growth under DS. NPs protect the photosynthetic apparatus and improve photosynthetic efficiency, accumulation of osmolytes, hormones, and phenolics, antioxidant activities, and gene expression, thus providing better resistance to plants against DS. In this review, we discuss the role of different metal-based NPs to mitigate DS in plants. We also highlighted various research gaps that should be filled in future research studies. This detailed review will be an excellent source of information for future researchers to adopt nanotechnology as an eco-friendly technique to improve drought tolerance.

## Introduction

The world’s population is expected to reach 9.6 billion by the end of 2050, which requires an increase of 70–100% in crop productivity to meet the needs of the rising population (Rodrigues et al., 2017; Alabdallah et al., 2021). However, increases in global warming and climate change, a reduction in fertile land, overuse of fertilizers and pesticides, and increases in the intensity of abiotic stresses cause substantial yield losses (Hasan et al., 2021a; Hasan et al., 2021b). As a result, the reduction in crop productivity poses a severe threat to global food security. Therefore, it is imperative that appropriate measures to eliminate the deleterious impacts of abiotic stresses on crops are taken to ensure global food security (Genc et al., 2019; Hasan et al., 2021a). Drought stress (DS) is severe abiotic stress that negatively affects crop growth and productivity globally (Seleiman et al., 2021). Successful crop productivity is a significant challenge in the presence of DS (Fathi and Tari, 2016). The severity and frequency of DS will increase in the future, which will pose serious threats to crop production (Chapman et al., 2021).

DS inhibits seeds germination, plant growth, physiological functioning, photosynthetic efficiency, and hormonal activities (Rasheed et al., 2022a; Shah et al., 2022). DS greatly affects the root morphology and spatial distribution of crops. DS, except in soils with high moisture content, affects root depth, length, and surface area (Chun et al., 2021). DS also reduces chlorophyll synthesis and increases the canopy temperature (CT), which causes a reduction in photosynthesis and plant metabolic activities (Morales et al., 2020). Water deficiency also reduces membrane permeability (**Table S1**) and increases reactive oxygen species (ROS) production (Rao and Chaitanya, 2019), which causes a deterioration in membrane integrity, increases electrolyte leakage (EL), and causes damage to DNA, proteins, and lipids (Shah et al., 2017). DS reduced seed yield by lowering photosynthesis, transpiration, and chlorophyll constituents (Mafakheri et al., 2010). In another study, Schittenhelm (2010) studied the impact of DS on the yield and quality of maize (*Zea mays*) and sorghum (*Sorghum bicolor*), and concluded that DS greatly affected the substrate composition in these crops (Schittenhelm, 2010). Plants have developed a built-in tendency to counter the effects of DS; nonetheless, they only show resilience to a certain extent (Shah et al., 2017; Shah et al., 2022). Plants accommodate DS by using various mechanisms, including accumulation of osmolytes, hormones, and activation of the antioxidant defense system (Hassan et al., 2020; Rasheed et al., 2022b). Climate change and the consequent frequency of DS have adversely affected crop productivity globally (Matuszak-Slamani et al., 2022). To increase crop productivity and counter the effects of DS, various strategies, including screening and breeding of tolerant cultivars, osmolytes, hormones, nutrient application, and microbes, can be used to increase crop production (Arslan et al., 2021). These strategies can protect the plants from DS and substantially increase crop productivity to meet food needs (Alabdallah et al., 2021).

Nanotechnology (NT) has emerged as a promising field and is commonly used in the agricultural, food, and medical industries (Alabdallah and Hasan, 2021). Various nanoparticles (NPs), including titanium dioxide (TiO_2_), iron oxide (Fe_3_O_4_), zinc oxide (ZnO), silicon oxide (SiO_2_), copper (Cu-NPs), and selenium (Se-NPs), have received significant attention recently owing to their non-threatening use in the agriculture sector (Hafeez et al., 2015; Alabdallah and Hasan, 2021; Hashem et al., 2021). Specific strategies, including chemical, green, and physical processes, can be used to produce NPs (Akhtar et al., 2022). NPs have positive impacts on plant growth and development; however, these effects can vary based on origin, size, concentration, and time of application to crops (Rubilar et al., 2013). Recently, NPs have improved plant tolerance against biotic and abiotic stresses. NPs protect plants from oxidative damage by increasing the activities of antioxidants (Ahmed et al., 2021). NPs can reduce drought-induced toxic effects by decreasing hydrogen peroxide (H_2_O_2_) and malondialdehyde (MDA) accumulation, and maintaining the efficiency of the photosynthetic apparatus (Adrees et al., 2020; Ahmed et al., 2021). NPs can also penetrate the plant chloroplast and reach the photo system-II (PS-II) reaction center, and increase transmission of electrons and light absorption in chloroplasts under DS, thereby improving photosynthetic efficiency and plant growth (Maity et al., 2018).

In addition to their commercial use and prevalence in various products, there is also concern about the toxicological and environmental impacts of NPs (Seabra and Durán, 2015). Their excessive use causes oxidative stress and physiological abnormalities in plants, resulting in decreased antioxidant activities and gas exchange characteristics (Wang et al., 2018). NPs also reduce the mitotic index and disturb the processes of cell division and root growth (Kumari et al., 2011). NPs also cause indirect toxicity by changing the growth medium and soil microbial activities (Ge et al., 2014). Many authors have reported the positive effects of NPs on growth under DS and a lack of understanding of the interaction of NPs and intercellular mechanisms in plants under DS. Therefore, in this review, we present information on different biochemical, physiological, and molecular mechanisms mediated by NPs to induce DS tolerance in plants. We also identify various research gaps that should be filled in future research studies for the use of NPs in the future. A better understanding of NPs and drought-stressed plants will open new opportunities to improve production in drought conditions. This comprehensive review will be valuable source of information to conduct more studies and develop more eco-friendly NPs of different metals to counter the devastating impact of DS on plants.

## Synthesis and characterization of metal oxide nanoparticles

**Figure 1:**
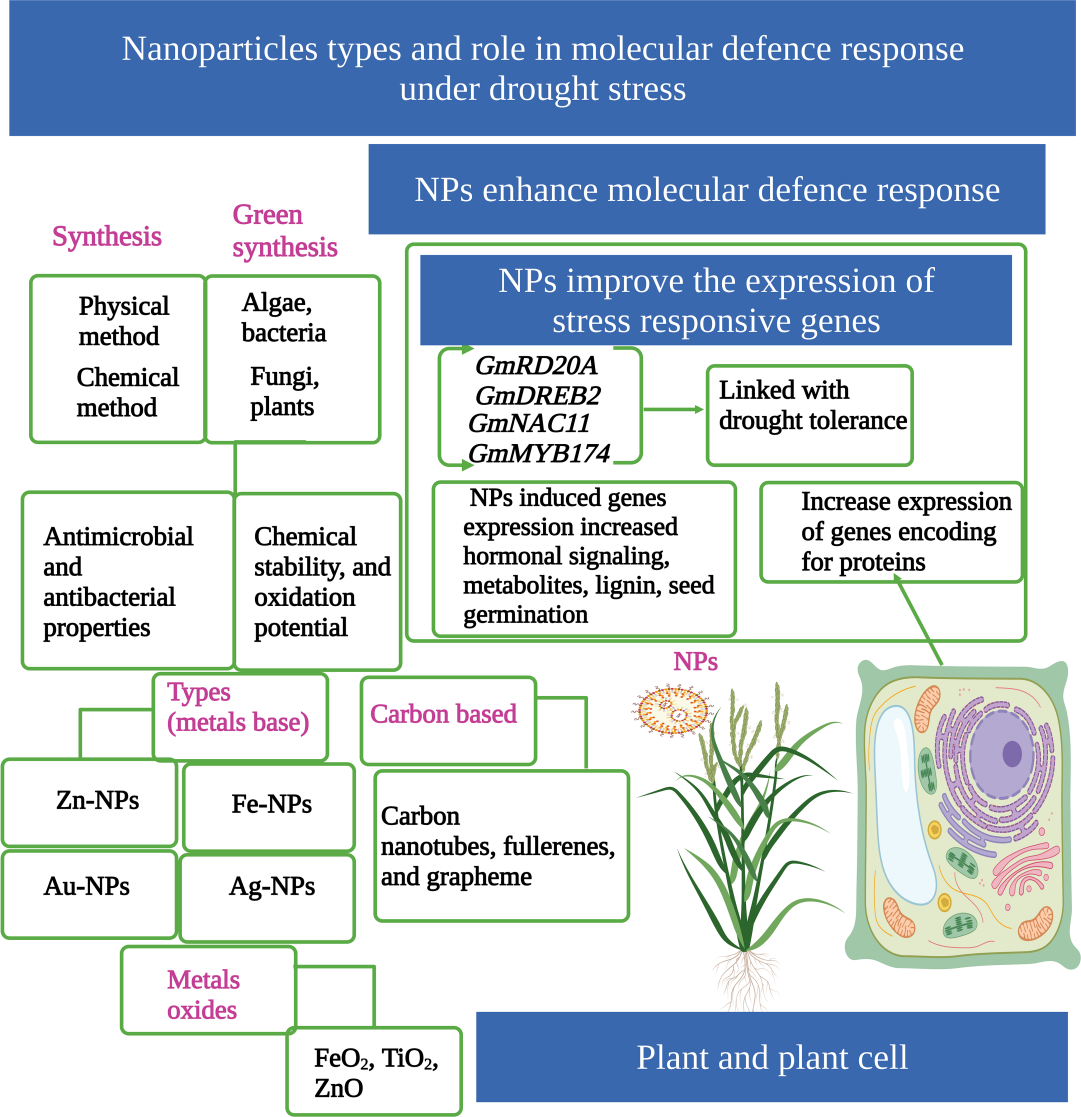
Nanoparticles (NPs) play a key role in drought tolerance in plants. NPs can be prepared by two main methods (physical or chemical). Green synthesis of NPs is an environmentally friendly technique. There are different groups of NPs, such as metal-based, metal-oxide-based, and carbon-based NPs. NPs increase the expression of genes under drought stress (DS) and protect plants from toxic effects. This Figure is created with BioRender.com.

The conventional methods for producing NPs are based on chemical and physical processes involving the use of dangerous and expensive substances, which have large energy requirements and adverse impacts on the environment (Alabdallah and Hasan, 2021). The green synthesis of NPs has recently attracted significant attention owing to their environmentally friendly nature (Durán and Seabra, 2012). Compared to conventional methods (chemical and physical), the green synthesis of NPs by various organisms (algae, bacteria, fungi, and plants) (**Figure 1**) provides an excellent environmentally friendly option (Durán and Seabra, 2012; Rubilar et al., 2013).

Green synthesized NPs have excellent photo-catalytic activity, chemical stability, and oxidation potential. Moreover, green synthesized NPs also have potent antimicrobial and antibacterial properties, which enable NPs use in a wide range of industries (Singh et al., 2016; Bahri et al., 2021). Zn-NPs have been extensively used in the formulation of cosmetics and sun-screen lotions, and they possess excellent antibacterial and anticancer properties (Dutta et al., 2012; Bogutska et al., 2013; Selvakumari et al., 2015). Zn-NPs are stable and affordable to synthesize and have shown an appreciable potential to improve crop production under abiotic stresses (Javed et al., 2017; Deka et al., 2019). Iron oxide NPs are also significantly used in biomedical fields, including cancer diagnosis, treatments, drug delivery, and nuclear magnetic resonance imaging (Seabra et al., 2014). Molybdenum (MO) is toxic at higher concentrations, and it has lower solubility in water, therefore can be used in high pressure and high temperature applications (Javed et al., 2020). MO-NPs can be produced by a minuscule amount of *in situ* radioactivity (Feng and Weicheng, 2016). MO-NPs can also be produced by a green synthesis approach. In this technique, the leaves of different plants can be taken and an extract prepared by standard procedures. After obtaining the extract, it is heated for 5–10 minutes and filtered, which can be used later to prepare the MO-NPs (Sreevani and Anierudhe, 2022). The green synthesis of MO-NPs is an environmentally friendly approach compared to chemical synthesis (Sreevani and Anierudhe, 2022).

Various capping agents and structural hosts used to limit the growth of NPs. Phenolic compounds are used as capping agents to improve colloidal stability and prevent the aggregation of NPs (Alabdallah et al., 2021). Tannins are a non-toxic and naturally occurring compound derived from plants. Tannins can be used to create NPs with appreciable potential to improve plant performance (Herrera-Becerra et al., 2010). Similarly, peel extracts from Plantago spp. and *Malus domestica*, as well as extracts from *Tridax procumbens*, also possess excellent potential as a capping agents and can be used to prepare NPs (Senthil and Ramesh, 2012; Venkateswarlu et al., 2013).

## Nanoparticle types and their mode of uptake in plants

The properties of NPs, such as electrical conductance, magnetism, chemical reactivity, optical effects, and physical strength, are different from bulky materials, owing to their large surface area and small size (Asha and Narain, 2020). Nano-materials are considered to form a link between their respective NPs and bulk materials (Boisseau and Loubaton, 2011). Different methods of NP preparation have been developed, which can be used to develop NPs of diverse shapes and sizes. Generally, two other processes (bottom-up and top-down) are used to create NPs. The bottom-up process creates NPs (SnS, MoS_2_, and Ags) from atoms and molecules, whereas the top-down approach creates NPs (CuO, MgO, ZnO, and graphite oxide (GO) from their macro-scale counterparts. The top-down technique involving the breaking of bulky materials into NPs, and this approach is an extension of the techniques being used for synthesis of micron-sized particles (Abid et al., 2021). Examples of this technique are high energy ball milling, atomic force manipulation, gas condensation, and aerosol sprays. On the other hand, the bottom-up technique refers to building material from the bottom: atom by atom, molecule by molecular, and cluster by cluster (Abid et al., 2021). Sol-gel synthesis, colloidal precipitation, template-assisted sol-gel, and electro-deposition are well known examples of bottom-up techniques to prepare NPs (Abid et al., 2021). Both top-down and bottom-up techniques are used to develop NPs around the globe, and each technique has its own advantages and disadvantages.

NPs can have one dimension (surface films), two dimensions (strands or fibers), or three dimensions (particles). Moreover, they can exist as spherical, tubular, or irregular shapes (Das and Das, 2019). NPs can be divided into four different groups; the first group is based on metal NPs (Au, Ag, Fe, Pt, Zn) (**Figure 1**), the second group is carbon-based NPs (carbon nanotubes, fullerenes, and grapheme), the third group is based on polymer compounds, and the fourth group is based on metal oxide NPs (FeO2, TiO_2_, ZnO) (**Figure 1**) (Paramo et al., 2020). The biological functioning of NPs depends on their concentrations, properties, and methods of application (Ali et al., 2021b). Different techniques, including seed treatments, foliar sprays, and soil application, are used to apply NPs to crops (Mahdy et al., 2020). NPs enter into plant tissue from the wounded regions and root junction; from these regions, they enter into cell walls and membranes and then move into plant leaves (Tripathi et al., 2017a). NPs also penetrate leaf cuticles, stomata, trichrome, hydathodes, and cell cytoplasm (Sharif et al., 2013). In cell cytoplasm, NPs bind with various organelles, interfering with different metabolic processes (Zhang and Monteiro-Riviere, 2009). NPs are also directly absorbed into the seed through the parenchymatic intercellular spaces in the seed coat (Tripathi et al., 2017b).

NPs’ accumulation, translocation, and uptake largely depend on plant species, size, and type of NPs, stability of NPs, and interaction of NPs with roots, soil, and soil microbes (Ali et al., 2021b). For instance, it has been noted that the accumulation of metal and metal oxide bases NPs by roots is significantly affected by the properties of the NPs and environmental conditions (Mittal et al., 2020; Ali et al., 2021b). For instance, it was noted that the application of Ag-NPs considerably increased the concentration of these NPs in the roots and shoots of lettuce plants (Doolette et al., 2015). It has also been recorded that root microbes significantly affected the mobility of NPs applied through foliar sprays, soil application, and root application (Feng et al., 2013). For instance, it was recorded that mycorrhizae reduced the uptake of Ag-NPs in the roots of *Trifolium repens* (Feng et al., 2013). Conversely, it was noted that the uptake of Se-NPs significantly increased owing to the presence of microbes (Durán et al., 2013).

Mucilage significantly affects the absorption of elements and the growth of soil microbes (Mckenzie et al., 2013). Mucilage acidifies the rhizospheric environment (Schaller et al., 2013), which as a result improves the uptake of metal-based, carbon-based, and all other types of NPs by plants (Schwab et al., 2016). The methods of application, climatic conditions, size, and concentration of NPs are the fundamental factors affecting the adsorption of NPs (Wang et al., 2013). Moreover, the morphology of leaves and the presence of trichomes, waxes, and exudates on the leaf surface also affects the absorption of NPs through the leaf surface (Larue et al., 2014). Furthermore, the presence of chemicals in pesticides also affects the absorption of NPs (Schwab et al., 2016).

Many studies noted that the size of the cell wall’s pores is a significant issue in the entry of NPs into the plant cell. Small NPs can pass directly into root epidermal cells (Ali et al., 2021a); however, plant roots’ epidermal cells are considered semi-permeable and contain tiny pores that restrict the entry of large NPs. Moreover, the leaf cuticle also acts as a barrier and restricts the access of NPs with a size of < 5 nm (Ali et al., 2021a). Generally, the basic structure of NPs plays the dominant role in the uptake and translocation of NPs (Raliya et al., 2016). Therefore, it is imperative that laboratory studies be performed to determine the exact impacts of NPs, considering their different physical and chemical characteristics (Zhang et al., 2012). More studies are also needed to understand the absorption, accumulation, and translocations of NPs in plant bodies. Moreover, it would be fascinating to determine the movement as well as localization of NPs to various structures, including cellular organelles, and to monitor and track NPs.

## Role of nanoparticles against drought stress

NPs enter the plant body through the roots and leaves, and after entering the plant body, NPs induce biochemical, morphological, molecular, and physiological changes in the crops (Khan et al., 2019). These changes significantly affect plant growth depending on the concentration, size, and application method of the NPs. Moreover, the size, chemical nature, and reactivity of NPs can substantially impact plants. The available evidence indicates that NPs mitigate the adverse effects of DS and appreciably improve plant growth and development (Ahmed et al., 2021).

## Nanoparticles improve membrane stability and plant–water relationships to confer drought stress

DS negatively affects cellular membranes and plant**–**water relationships, causing a significant reduction in plant growth (Hassan et al., 2020). DS induced ROS production, which damages cell membranes, causes lipid peroxidation, and increases MDA accumulation (Das and Roychoudhury, 2014). The application of NPs (ZnO) significantly decreased the MDA and H_2_O_2_ accumulation and maintained membrane stability which reduced the loss of essential osmolytes (Mohamed et al., 2017; Sun et al., 2020; El-Zohri et al., 2021). Water deficiency substantially reduced membrane stability, efficiency of PS-II, and chlorophyll contents (Semida et al., 2021). However, the exogenous application of NPs (ZnO) maintained membrane stability and cell water status under DS, thereby improving efficiency of PS-II and metabolic processes (Yan et al., 2016; Zhao et al., 2017; Semida et al., 2021).

The application of NPs improved plants’ anatomical features, which in turn maintained membrane stability and cell stability, thereby ensuring better water uptake (Yan et al., 2016; Hafez et al., 2020). The application of NPs (ZnO) induces changes in root morphology and increases the formation of lateral roots and root biomass, consequently improving water uptake and maintaining better water status under DS (Ahmad et al., 2017; Behboudi et al., 2018; Alsaeedi et al., 2019; Dimkpa et al., 2019; Zahedi et al., 2020a). Moreover, in another study, it was noted that SiO_2_ NPs significantly reduced the negative impacts of DS by increasing photosynthesis (**Table S2**), transpiration, relative water content (RWC), and water uptake (Sutulienė et al., 2021). Additionally, NPs also increase root hydraulic conductivity, gene expression, and stress and hormonal signaling, which allows better water uptake and thereby ensures better plant**–**water status under DS (Das and Das, 2019). NPs also increase expression of aquaporins, maintain hydraulic pressure, increase root length, and allow better root penetration, allowing better water uptake by plants (Faraji and Sepehri, 2020). In conclusion, NP-based maintenance of membrane stability and plant**–**water relationship can reduce the adverse effects of DS. Nonetheless, the effect of NPs on membrane compositions, osmolyte accumulation, and transportation of different solutes across the membrane needs further study to better understand the role of NPs against DS.

## Nanoparticles improve nutrient uptake under drought stress

DS significantly disturbs nutrient homeostasis and induces nutrient deficiency, which adversely affect plant growth (Hassan et al., 2020). NPs play a significant role in nutrient homeostasis and significantly improve nutrient uptake, translocation, and allocation to different plant parts (Kopittke et al., 2019). Water deficiency reduced the concentration of nitrogen (N), potassium (K), manganese (Mn), and Zn in plants (Semida et al., 2021), which was a consequence of a reduction in nutrient uptake, transpiration flux, and membrane stability (Dimkpa et al., 2017). The application of NPs (ZnO) through soil and applied by foliar spray significantly improved the uptake of N, phosphorous (P), K, and Zn, and attenuated the adverse effects of DS (Mangena, 2018; Dimkpa et al., 2019; Fatollahpour Grangah et al., 2020; Mustafa et al., 2021; Semida et al., 2021; Akhtar et al., 2022).

Ahmed et al. (2021) noted that Fe- and Hg-NPs significantly increased N (51%), P (61%), K (27%), calcium (Ca) (53%), and magnesium (Mg) (62%) uptake compared to the control. The combination of Si-NPs and plant growth-promoting rhizobacteria (PGPR) significantly improved photosynthesis, RWC, antioxidant activities, and nutrient uptake in maize plants grown under DS (Hafez et al., 2021). Researchers from Iraq also noted that applying NPs in combination with bio-fertilizers significantly improved nutrient uptake, nodule formation, and nitrogenase activity of *P. vulgaris* (Al-Burki and Al-Ajeel, 2021). Wheat and sorghum plants treated with Zn-NPs showed a significant improvement in productivity and nutrient uptake (Dimkpa et al., 2017; Dimkpa et al., 2019). The application NPs significantly improved the uptake of nutrients, nitrate reductase activity, and assimilation of N, which in turn improved the synthesis of proteins and amino acids (Yuan et al., 2013). The application of NPs enhances the sequestration of nutrients to plant roots and increases nutrient uptake by plants (Jaberzadeh et al., 2013). Additionally, the use of NPs increases water use efficiency, which also results in substantial improvements in nutrient uptake and plant growth (Jaberzadeh et al., 2013). In conclusion, the use of NPs in plants improves nutrient uptake, plant growth, and DS tolerance. Nonetheless, the role of NPs in nutrient signaling and nutrient channels should be explored for future possibilities.

## Nanoparticles protect photosynthetic apparatus and improve photosynthesis under drought stress

Water deficiency decreases chlorophyll synthesis, PS-II efficiency, and electron flow rate, thereby negatively affecting overall plant photosynthetic efficiency (Semida et al., 2021). Applying NPs (ZnO) improved the synthesis of chlorophyll, chlorophyll fluorescence, and activity of chlorophyll synthesis enzymes (chlorophyllase), which in turn improved the photosynthetic efficiency under DS (El-Mageed et al., 2021; Semida et al., 2021). The exogenous application of NPs also stabilizes the ultra-structure of chloroplast and mitochondria, which helps plants to maintain their photosynthetic efficiency under DS (Rahmatpour et al., 2018).

**Figure 2:**
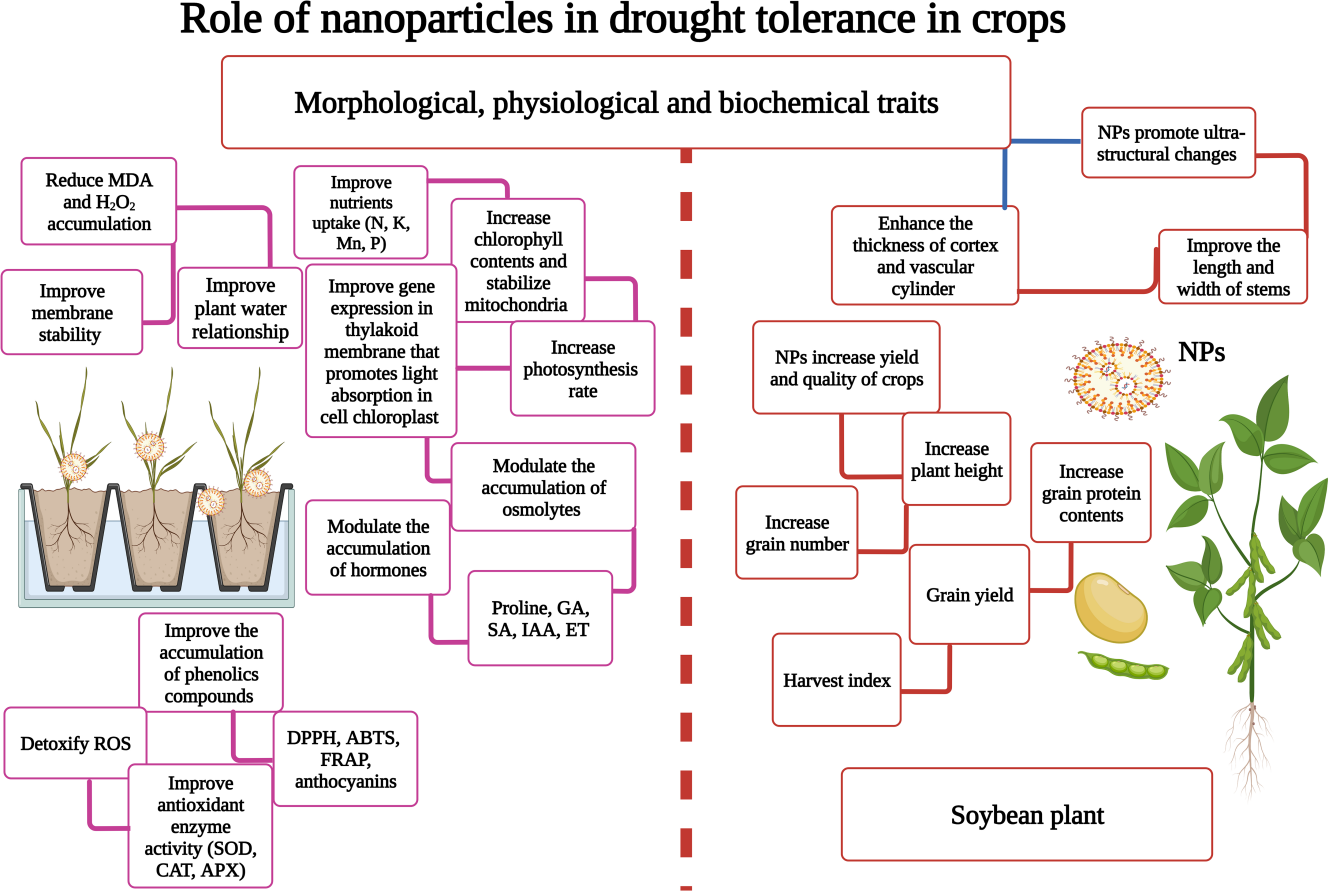
Nanoparticles (NPs) play a key role in enhancing drought stress (DS) tolerance in plants. NPs reduce MDA accumulation, maintain membrane stability, induce the expression of stress-related proteins, improve nutrient and water uptake, increase photosynthesis, and increase grain yield and harvest index. This Figure is created with BioRender.com.

The application of NPs (TiO_2_) increases light-induced water hydrolyzation into oxygen, electrons, and protons (Silva et al., 2022), which ensures the entry of electrons and protons into the electron transport chain and results in a considerable increase in plant photosynthetic efficiency (Alabdallah et al., 2021). In addition, NPs (TiO_2_) also improve the expression of genes (LHCII-b) in the thylakoid membrane, which promotes light absorption in cell chloroplast (**Figure 2**) (Ze et al., 2011). Moreover, NPs (TiO_2_) also improve Ribulose-1,5-bisphosphate carboxylase/oxygenase (RuBisCO) activity, nitrogen assimilation, and nitrate reductase activity, which in turn ensure better photosynthetic performance under DS and improve photosynthetic efficiency in plants under DS (Yuan et al., 2013; Ali et al., 2021a).

Stomata control the exchange of water vapors and CO_2_ between the atmosphere and leaf interior surface (Suzuki et al., 2013). DS also activates the ABA signaling pathway that induces stomata closure (Sierla et al., 2016; Faraji and Sepehri, 2020; however, application of NPs down-regulated the H_2_O_2_-mediated stomata closing and maintained better CO_2_ intake (Djanaguiraman et al., 2018). The application of NPs (TiO_2_) improves photosynthetic pigments and gas exchange characteristics by increasing the activities of enzymes in CO_2_ fixation and synthesis of chlorophyll (Mohammadi et al., 2016; Faraji and Sepehri, 2020).

NPs also improve light absorption in chloroplast and enhance electron transport, efficiency of PS-II, O_2_ evolution, and photo-phosphorylation, improving the plant photosynthetic efficiency under DS (Shafea et al., 2017). NPs also increased the uptake of Ca, Mg, N, K, and iron (Fe), and improved gas exchange characteristics, which ensured better photosynthesis under DS (Faraji and Sepehri, 2020). Carotenoid works as an antioxidant to protect the chlorophyll from oxidative damage under stress conditions (Emiliani et al., 2018). Applying NPs (CuO) significantly improves carotenoid contents, which protect the chlorophyll from degradation and enhance the chlorophyll concentration, thereby improving photosynthetic efficiency under DS (Van Nguyen et al., 2022). In conclusion, the application of NPs is an effective technique that improves photosynthesis by increasing nutrient uptake, and improving chlorophyll synthesis and efficiency of PS-II.

## Nanoparticles improve osmolyte accumulation and maintain hormonal crosstalk to confer drought tolerance

NPs modulate the accumulation of osmolytes and hormones that reduce oxidative stress by strengthening antioxidant machinery (Silva et al., 2022). Proline accumulation improves plant**–**water status, stabilizes proteins, DNA, enzymes, and cellular membranes, and scavenges the ROS, protecting plants from drought-induced oxidative damage (Farooq et al., 2017). The application of NPs (TiO_2_) induces the *P5CS1* gene encoding d1-pyrroline-5-carboxylate synthetase, which is involved in proline synthesis (Mohammadi et al., 2016). NPs up-regulate the synthesis of proline and sugars that maintain cellular membranes, proteins, and enzymes under DS, thereby improving plant performance under DS (Gohari et al., 2020; Zhang et al., 2020). Similarly, NPs (TiO_2_) also appreciably improved the accumulation of proline, soluble sugars, total proteins, glycine betaine, and phenolic compounds, and improve plant growth under DS (Kardavan Ghabel and Karamian, 2020; Mustafa et al., 2021).

The application of NPs increases the synthesis of indole acetic acid (IAA) and gibberellins (GA), which in turn improve plant growth under DS (Churilova et al., 2017; Li et al., 2021). The application of Fe-NPs greatly enhanced the growth and yield of strawberry (*Fragaria × ananassa* Duch.) in combination with salicylic acid (SA), which is a significant growth hormone in plants (Havas and Ghaderi, 2018). Sun et al. (2020) noted that NPs induced enhanced drought tolerance by increasing melatonin synthesis, which indicates the role of NPs in combating DS in crops by increasing endogenous hormones. Mustafa et al. (2021) noted that applying 40 ppm TiO_2_-NPs increased IAA and GA levels by 49.07% and 27.43% respectively, and decreased ABA levels by 31.32% (Mustafa et al., 2021). In conclusion, NP-mediated maintenance of hormonal balance can improve drought tolerance in plants. The effect of NPs on the synthesis and concentration of several plant hormones like cytokinin (CK), ethylene (ET), and ABA has not yet been explored; therefore, it is imperative that more studies are undertaken to explore the role of NPs in the synthesis of these hormones.

## Nanoparticles improve the accumulation of phenolics compounds under drought stress

DS significantly reduces the accumulation of phenolic compounds; however, NPs possess an excellent potential to improve their accumulation in plants. The exogenous supply of ZnO-NPs (25 and 50 mg/L) significantly enhanced the concentration of phenolic compounds under DS (El-Zohri et al., 2021), which in turn increased the antioxidant activities and thereby reduced MDA and H_2_O_2_ accumulation (El-Zohri et al., 2021). Javed et al. (2017) investigated the impact of ZnO-NPs on phenolic contents of *Stevia rebaudiana*, and they noted that ZnO-NPs (100 and 1000 mg/L) significantly reduced phenolic concentration. In addition, it has been reported that total phenolic compounds and non-enzymatic activities (DPPH, ABTS, and FRAP) (**Figure 2**) significantly increased in NP-treated plants, reducing oxidative damage by decreasing lipid peroxidation (Sun et al., 2021; Ghani et al., 2022).

Anthocyanins are a group of phenolic compounds with excellent antioxidant properties (Hoekstra et al., 2001). Similarly, polyphenol compounds also have tremendous antioxidant potential (Posmyk et al., 2009). The application of Si- and Se-NPs showed a marked increase in anthocyanin, phenolic, and antioxidant activity (Zahedi et al., 2019; Zahedi et al., 2020a; Zahedi et al., 2021). Similarly, application of NPs (TiO_2_) also increases gene expression involved in the production of enzymes linked with syntheses of syringic, sinapic, and ferulic acids (Shoarian et al., 2020).

## Nanoparticles detoxify ROS and strengthen the antioxidant defense system under drought stress

The deficiency of water causes oxidative stress by increasing the accumulation of MDA, H_2_O_2,_ and ROS (Sutulienė et al., 2021). NPs possess an excellent potential to improve antioxidant activity to mitigate the detrimental impacts of DS. The application of NPs (ZnO, Se, and Si) significantly improved APX, CAT, and SOD activity (**Figure 2**), which reduced drought-induced oxidative damage (Hernandez-Viezcas et al., 2011; Khan et al., 2017; Venkatachalam et al., 2017; Zahedi et al., 2020b; Ali et al., 2021a; Sutulienė et al., 2021). In the same context, NPs also increase the relative abundance of Cu/Zn-SOD, APX, and CAT (**Table S3**) under DS, which noticeably scavenge the ROS (Sun et al., 2020). The application of NPs (ZnO) also improves non-enzymatic antioxidant activities (phenolic compounds, AsA) that work in coordination with antioxidant enzymes (APX, CAT, and SOD) (El-Zohri et al., 2021). NPs (SiO_2_) significantly increased non-enzymatic activities by increasing TPC, DPPH, and FRAPS activity in plants grown under DS (Miller and Rice-Evans, 1997; Sutulienė et al., 2021). NPs trigger the accumulation of antioxidant genes, osmolytes, nutrients, and amino acids, which increases antioxidant activities and thus protects the plants from oxidative stress (Mittal et al., 2020).

The use of NPs (TiO_2_) also increases nitrate reductase activity and accumulation of osmolytes. NP-mediated increases in NR activity leads to NO formation being induced, which stimulates proline and glycine betaine (GB) synthesis, and protects the plants from oxidative damage (Khan et al., 2020). NPs also significantly increases the accumulation of glucose, fructose, trehalose, and sucrose, which increase antioxidant activities and, as a result, improve drought tolerance (Heikal et al., 2022). NP-mediated improvements in antioxidant activities mitigate the adverse effects of DS on plants by scavenging the ROS.

## Nanoparticles improve the expression of stress-responsive genes under drought stress

Drought responsive genes including *GmRD20A*, *GmDREB2*, *GmERD1*, *GmFDL19*, *GmNAC11*, *GmWRKY27*, *GmMYB118*, and *GmMYB174* and their expressions are significantly up-regulated by the application of NPs (Kandhol et al., 2022) (**Figure 1**). In another study, Yang et al. (2018) also noted that CuO-NPs and ZnO-NPs significantly increased the expression of genes linked with drought tolerance in the roots of wheat plants (Yang et al., 2018). Moreover, CS-NPs up-regulated the expression of genes linked with alkaloid biosynthesis and increased the antioxidant potential of *Catharanthus roseus* grown under DS (Ali et al., 2021a). The up-regulation of deacetylvindoline-4-Oacetyltransferase (DAT), strictosidine synthase (STR), peroxidase 1 (PRX1), and geissoschizine synthase (GS) genes following application of CS-NPs increased the concentration of alkaloid contents (Vakilian, 2019).

Ag-NPs increased the expression of genes associated with IAA, 9-cis carotenoid dioxygenase (NCED3), and RD22 proteins in response to DS, and they also suppressed the ACC 7 synthase (ACS7) and ACC oxidase 2 genes in *Arabidopsis thaliana* grown under DS (Pérez-Labrada et al., 2020). Similarly, Cu-NPs increased the process of glycolysis, the tricarboxylic acid cycle, and starch degradation mechanism, leading to significant improvements in drought tolerance (Yasmeen et al., 2017). Additionally, ceria-based NPs significantly increased the protein expression associated with stress tolerance and down-regulated the proteins involved in the storage of nutrients and carbohydrate metabolism in kidney beans (Majumdar et al., 2015).

NPs also increased the expression of *GmWRKY27*, *GmMYB118*, and *GmMYB174*, which increased hormonal signaling, synthesis of secondary metabolites, lignin, seed germination, and plant responses against DS (Rushton et al., 2010). The higher expression of the *GmWRKY27* gene in NP-treated plants suggested that NPs are linked with regulation of ABA biosynthesis and functioning of stomata under DS (Linh et al., 2020). Likewise, Fe- and Co-NPs significantly increased the expression of *GmRD20A*, suggesting that NPs induce Ca^2+^-binding protein expression under DS (Linh et al., 2020). Recently, Sun et al. (2020) also noted that Zn-NPs increased the expression of Fe/Mn-SOD, Cu/Zn SOD, APX, and CAT, which improved drought tolerance. Yalda Raeesi Sadati et al. (2021) studied the expression pattern of some transcription factors (TFs) in wheat under DS, and the application of Zn-NPs significantly increased the expression of *WRKY1*, *HMA2*, and *ZIP1* genes, resulting in a substantial increase in drought tolerance. In conclusion, the application of NPs increased gene expression which in turn increased hormonal signaling, synthesis of secondary metabolites, and antioxidant activities, thereby improving drought tolerance in plants. Nonetheless, the expression of NP-based activation genes has not been studied in depth, and more research is necessary to explore the mechanisms behind increases in drought tolerance following increases in gene expression after the application of NPs.

## NPs bring ultra-structural changes to induce drought tolerance

Applying Zn-NPs (100 ppm) clearly improved the length and width of stem and vascular cylinder, and increased the thickness of the cortex and vascular cylinder (Semida et al., 2021). The plant processes, including physiological, biochemical, and anatomical mechanisms, are responsible for the performance of plants, and these functions are linked to a plant’s internal anatomy (Petrov et al., 2018). Application of NPs improved anatomical parameters, RWC, membrane stability, and nutrient status (Hafez et al., 2020). Water deficiency decreases vessel diameter and vascular length and width. A foliar spray of Zn-NPs significantly increased the thickness of the epidermis, the size and width of the vascular bundle, and the diameter of the vessel, which improved drought tolerance (Al-Dhalimi and Al-Ajeel, 2020). There is limited information available in the literature regarding the role of NPs on plant ultra-structural changes under DS. Therefore, more studies are urgently required to investigate the role of NPs on these aspects under DS.

## Nanoparticles improve growth, yield, and quality under drought stress

The primary response of DS is stomata closing, which affects CO_2_ diffusion, reduces photosynthesis, and diminishes plant growth (Abdelkhalik et al., 2019). The application of NPs improves plant growth by triggering hormonal signaling, root activity, water uptake, and antioxidant activities (Ahmad et al., 2017). A foliar spray of NPs improves photosynthetic efficiency, synthesis of secondary metabolites and chlorophyll, and antioxidant (APX and SOD) activity (**Table S4**), thereby improving plant growth under DS (Djanaguiraman et al., 2018; Zahedi et al., 2019; Semida et al., 2021; Van Nguyen et al., 2022). The soil and foliar application CS-NPs (60 and 90 ppm) reversed the adverse effects of DS and improved the yield and yield components compared to the control (Behboudi et al., 2018). CS-NPs have a positive ionic charge and ensure slow nutrient release in plants, which improves grain weight, plant height (PH), and harvest index (HI) under DS (Abdel-Aziz et al., 2016).

NPs also improve water and nutrient uptake and reduce the production of harmful free radicals by increasing antioxidant activities that can boost plant growth under DS (Behboudi et al., 2018). The application of NPs also significantly improved the quality of crops grown under DS. Likewise, it has been recorded that the application of NPs led to a maximum concentration of grain protein contents compared to the control (Behboudi et al., 2018). The application of NPs increases the N uptake, which improves protein synthesis in plants under DS (Hatami and Ghorbanpour, 2014; Behboudi et al., 2018). DS stress reduces protein and starch contents, and it has been reported that the application of Zn-NPs improved the starch and protein contents under DS (Waqas Mazhar et al., 2022). In another study, applying Zn-NPs enhanced the amylase activity, increasing nutrient uptake and mobilization (Do Espirito Santo Pereira et al., 2021). Itroutwar et al. (2020) noted that ZnO-NPs applied by seed priming markedly improved rice bio-fortification grown under DS (Itroutwar et al., 2020). The experiments conducted by Yasmeen et al. (2017) showed that Fe- and Cu-NPs significantly improved the spike length, grain/spike, and grain weight of wheat plants grown under DS. Similarly, in rice plants, ZnO-NPs also increased nutrient uptake by increasing the synthesis of enzymes involved in nutrient uptake and acquisition (Khanra et al., 2018). Growth, yield, and quality are the most significant parameters of any crop badly affected by DS. NP-mediated yield and quality enhancement is linked with improved antioxidant activity, photosynthetic efficiency, and nutrient and water uptake.

## Toxic effects of nanoparticles

NPs interact with plants by various chemical and physical means, and these interactions cause various signaling that leads to the production of ROS. NP-mediated increases in ROS production can cause damage to plant cells and substantially reduce the plant growth and development (Nel et al., 2006; Xie et al., 2019). The toxic effects of NPs depend on the size, shape, and properties of the NPs. Certain metal NPs, including iron, silver, platinum, and gold, and metal oxide NPs, including Fe_3_O_4_, ZnO, and TiO_2_, used in various sectors can be dangerous to human health. These NPs come into contact with cells and damage protein, DNA, and membranes, and can induce a significant reduction in plant growth (Hsin et al., 2008). These NPs can also enter into the blood stream and reach vital organs where they can cause serious toxicity (Hsin et al., 2008). Soil is considered a large reservoir of NPs, and plant roots absorb NPs and nutrients from soil by active transport (Khan et al., 2021). After being absorbed by the roots, NPs infiltrate into the epidermis of cell walls and root cortex, and then move to upper parts of the plant where they can cause serious toxicity (Rajput et al., 2018). At higher concentrations, NPs also affect plant growth by decreasing chlorophyll synthesis, photosynthetic performance, and antioxidant activities (Wang et al., 2018). NPs, being small in size, get absorbed into biological systems around 15–20 times faster compared to conventional bulky materials (Khan et al., 2021). NPs are absorbed by soil systems, and they adversely affect natural fauna, including bacteria, fungi, and nematodes (Handy and Shaw, 2007; Khan et al., 2021). Studies indicated that NP-mediated toxicity in plants, algae, and other microbes is associated with physical damage and oxidative stress generated by ROS production (Hou et al., 2018).

In addition to plants, NPs cause cyto- and genotoxicity in mammals because of the production of ROS (Khan et al., 2015). NPs also enter into the environment through discharge from nano-powered items, discharge during use, and discharge after removal of NPs from materials containing them (Gottschalk et al., 2013; Tolaymat et al., 2017). Likewise, in aquatic environments, NPs also change photosynthetic color structure, efficiency of PS-II, and growth of amphibian plants (Jacobasch et al., 2014). Nanotechnology has emerged as an important tool to improve energy consumption efficiency and environmental health, and to solve various health problems. It is considered an important approach that can increase manufacturing production at reduced costs (Khan et al., 2021). NPs can be produced by physical, chemical, biological, and mechanical means, and each method of synthesis has its own advantages and disadvantages. The physical method of NP synthesis is expensive, whereas chemical methods can pose environmental risks along with slow growth rates (Pandey and Jain, 2020). The biological method of NP synthesis is environmentally friendly and non-toxic, and this eco-friendly approach is more acceptable than traditional methods (Patra and Baek, 2014).

## Conclusion and future perspectives

DS significantly reduces plant growth and development by disturbing the plant’s biochemical, molecular, and physiological processes. However, the use of NPs substantially improves plant performance and provides substantial resistance to plants against DS. The use of NPs enhances the stability of membranes and nutrient and water uptake, and protects the plant photosynthetic apparatus from the damage caused by DS, thereby improving plant growth under DS. The application of NPs also enhances the accumulation of stress-protective hormones, osmolytes, and phenolics. Moreover, NPs also enhance the expression of stress-responsive and antioxidant genes, leading to significantly improved mechanisms against DS. In recent years, the role of NPs in mediating the various mechanisms to induce DS tolerance has been well explored.

The role of NPs in seed germination has not yet been studied; therefore, it is necessary that the role of NPs in germination mechanisms is examined, including water uptake by seeds, radical protrusion, and activation of enzymes involved in food mobilization. In addition, the role of NPs on the metabolic aspects of gibberellin and abscisic acids should be explored because these hormones are imperative in seed germination. The application of NPs improves nutrient uptake under DS; however, the role of NPs on nutrient channels and ionic transporters in plants under DS should be explored. The application of NPs significantly protects photosynthetic apparatus; however, the role of NPs on intercellular signaling, stomata movements, and regulation of anion channels in guard cells of plants under DS should also be explored.

The role of NPs in osmolytes and hormone accumulation has been well studied; however, more studies are needed to explore the role of NPs in accumulation hormones and osmolytes in plants under DS. The effect of NPs on plants’ ABA-dependent and independent responses to DS should also be studied in detail. The effect of NPs on salicylic acid, gibberellic acid, cytokinin, ethylene, proline, and glycine-betaine should be explored at the transcriptomic level. Moreover, the role of NPs on gene expression, and enzymes linked with the synthesis of these osmolytes, should also be explored. It would also be interesting to determine the role of NPs in crosstalk of different hormones and osmolytes: this research gap should be filled. Additionally, identification as well as characterization of NP-mediated gene expression involved in hormone-mediated stimulation of osmolyte biosynthetic pathways will open new research directions.

The role of NPs in defensive and antioxidant genes should also be studied to increase our knowledge of inducing DS tolerance in plants. There is no information available about the metabolically active roles of NPs under DS; therefore, this area should be explored. Different types of NP transporters for the uptake of NPs have been identified in plants; however, the various transporters and channel proteins responsible for the loading of NPs have not yet been explored. Therefore, it is necessary that this role of NPs be explored in future research programs. It would also be fascinating to study the distribution of NPs in cell walls and cell nuclei to enhance tolerance against DS.

The effect of NPs on proteomics would also be worthwhile in order to increase our understanding of the different mechanisms mediated by NPs to induce DS tolerance. Morphological as well as physiological proteomic studies on NP- induced toxicity would determine the particle size and NP chemistry and concentration required for each plant species to ascertain the level of plant response against DS. In addition, more studies are required to establish whether NPs exert toxicity because of their large surface area, size, and release of metal ions. Additionally, -omic techniques, including integration genomics, transcriptomics, proteomics, and metabolomics, are also needed to determine the impact of NPs on plants.

Studies on the effect of NPs on proteomics would also be worthwhile to increase our understanding of the different mechanisms mediated by NPs to induce DS tolerance. The effects of NPs on proteomics and genetic factors have been poorly studied, and it would be beneficial to explore these aspects in future studies. In addition, combining microbes and NPs to induce DS tolerance would also be an attractive area of research. Moreover, developing detailed knowledge about the interactions of NPs and plants would facilitate a better understanding of DS tolerance in plants. Research is necessary to optimize the timing and concentration of NPs under diverse climate conditions for different crops. The majority of studies concentrate on the impact of NPs on plants under DS; however, as plants often face multiple stresses during their growth cycle, exploring the effects of NPs on plants under a combination of different stresses is necessary.

## Author contributions

AR conceptualized and prepared the manuscript. HL, MMT, AM, MN, ANS, MN, MTA, SN, MM and MUH reviewed and editing. ZW supervised the study.

## Funding

'The research was supported by the National Natural Science Foundation of China (31760350 and 71963020), the Training Program for Academic and Technical Leaders in Major Discipline in Jiangxi Province (20204BCJL22044), the Natural Science Foundation of Jiangxi (20202BABL205020), the Key Research and Development Program of Jiangxi Province (20192ACB60003), and the Jiangxi Agriculture Research System (JXARS-18).

## Acknowledgments

The authors are thankful to ZW for his supervision and support during the entire research work. The authors are also thankful to Dr. Muhammad Aamer for his valuable suggestions to improve the quality of the manuscript. The authors would also like to thank the Deanship of Scientific Research at King Khalid University, Abha, KSA for supporting this work under grant number (R.G.P.2/197/43).

## Conflict of interest

The authors declare that the research was conducted in the absence of any commercial or financial relationships that could be construed as a potential conflict of interest.

## Publisher’s note

All claims expressed in this article are solely those of the authors and do not necessarily represent those of their affiliated organizations, or those of the publisher, the editors and the reviewers. Any product that may be evaluated in this article, or claim that may be made by its manufacturer, is not guaranteed or endorsed by the publisher.
