# Supplementary material for: The role of nanoparticles in plant biochemical, physiological, and molecular responses under drought stress: A review
Source: Front Plant Sci. 2022 Nov 24;13:976179. doi: 10.3389/fpls.2022.976179 (PMC9730289; doi:10.3389/fpls.2022.976179)
Supplement: Supplementary file 1 [file DataSheet_1.docx]

**Table S1: Effect of drought stress on growth, physiological and biochemical process of various crops**

| **Crop species** | **Drought stress** | **Effects** | **References** |
| --- | --- | --- | --- |
| Pea leaves | -1.3 to -1.9 MPa | DS reduces the relative water contents (RWC), stomatal conductance, transpiration rate, chlorophyll contents (a and b) that affect photosynthetic activity which decreases the plant growth. | ([Bahadur et al., 2011](#_ENREF_23)) |
| Brant's oak | 20% FC | Net photosynthesis, stomatal conductance, Chlorophyll (a+b) and carotenoids contents decreases with increase in proline and soluble sugar concentration under severe DS that reduces plant growth (plant height, stem diameter and TDM) attributes. | ([Jafarnia et al., 2018](#_ENREF_85)) |
| Rapeseed | 15% PEG-6000 | Germination percentage, vigor index, RWC, shoot and root length along with their fresh and dry weight, total chlorophyll (a and b) and carotenoid contents were decreased with increasing ROS (MDA, protein, total soluble sugar, proline) under DS. | ([Batool et al., 2022](#_ENREF_25)) |
| Corn | 110 mm | RWC, number of grains/cobs, 100 grain weight, corn fresh weight and biological yield was significantly reduced under drought stress in which the increased level of proline contents was observed. | ([Noein and Soleymani, 2022](#_ENREF_118)) |
| Wheat | 40% FC | DS significantly declined the leaf characteristics (number and area), relative water content and leaf chlorophyll content while increased proline accumulation that reduces the grain and its contributing parameters under DS. | ([Mannan et al., 2022](#_ENREF_106)) |
| Urad bean | Dewatering at 13 days | DS significantly increased root length, decreased leaf area and shoot length while closure of stomata due to less RWC which trigger the production of ROS (ABA, CAT). Moreover, production of chlorophyll and carotenoid contents were increased under DS. | ([Gurumurthy et al., 2019](#_ENREF_65)) |
| Pot marigold | 60% FC | Biomass attributes (number of leaves and leaf area), photosynthetic pigments (chl a and b along with carotenoids), leaf water contents, relative turgidity, excised leaf water loss and retention along with membrane stability was negatively affected by drought. Moreover, antioxidants (SOD, CAT and GPX) produced in response of ROS production. | ([Akhtar et al., 2022](#_ENREF_10)) |
| Wheat | 50% FC | DS lowered plant growth parameters (plant height, root length, number of tillers/plants, leaves number and flag leaf area), fresh and dry weight of root and shoot along with chlorophyll a and b contents while increasing carotenoids, soluble sugars, free amino acids, proline, total phenols, and flavonoids. | ([Hussein et al., 2022](#_ENREF_80)) |
| Maize | 40% FC | Plant growth attributes (plant height, root, stem, leaves and shoot fresh weight), chlorophyll pigments, fluorescence was comparably reduced in DS, whereas, antioxidant activities were increasingly observed. | ([Ahmad et al., 2022](#_ENREF_7)) |

**Table S2: Effect of Nano-particles (NPs) on growth, and physiological attributes under drought stress**

| **Crop** | **Drought stress** | **NPs type** | **NPs concentration** | **Effects** | **References** |
| --- | --- | --- | --- | --- | --- |
| Rice | 35% WHC of soil with distilled water | ZnONPs | 25ppm | Nano-particles significantly increased the number of tillers, panicle per plant, seed, and straw yield, 1000 paddy weight, proline and A.A. contents, SOD, POD, CAT activities while reducing H_2_O_2_ and MDA concentration under DS. | ([Waqas Mazhar et al., 2022](#_ENREF_180)) |
| Cucumber | 2.5% NaClO | ZnONPs | 100 mg L^−1^ | Plant shoot and root weight (fresh and dry), photosynthetic pigments (chlorophyll a, b, total chlorophyll, and carotenoid contents) osmolytes (Pro, sugar and free A.A.) and antioxidants (SOD, POD, CAT, APX, DHAR, MDHAR, GR, GSH and ASA) production was increased while reducing EC and ROS (H_2_O_2_, O_2_^−^_,_ MDA) production under severe DS. | ([Ghani et al., 2022](#_ENREF_59)) |
| Maize | 21 days | (nano-Cu^o^) | 69.4 µM nano-Cu^o^ | Singlet Cu enhanced drought tolerance by protecting the leaf water content, anthocyanin, chlorophyll, and carotenoid contents. Moreover, it also increased plant biomass, total seed number and grain yield of maize under DS. | ([Van Nguyen et al., 2022](#_ENREF_174)) |
| Wheat | 15 days | ZnO-NPs | 10 ppm of ZnO-NPs | NPs improved wheat growth, biomass, protect photosynthetic pigments (chl a, b and carotenoids), trigger the production of antioxidants (proline, antioxidant enzymes i. e superoxide dismutase, peroxidase) in response to ROS production under DS. | ([Azmat et al., 2022](#_ENREF_22)) |
| Wheat | 35% FC | Se NPs | 30 mg L^-1^ | NPs significantly increased the plant height, length, fresh and dry weight of roots and shoots along with increasing number, length, and area of leaves under DS. | ([Ikram et al., 2020](#_ENREF_81)) |
| Marigold | −1.5 MPa | Si NPs | 0, 100, 200, 500 mg L^-1^ | NPs positively regulated seed germination, seedling length, dry weight, and vigor index under DS conditions. | ([Rahimi et al., 2021](#_ENREF_127)) |
| Wheat | 2 weeks | Fe_3_O_2_-NPs | 1.2 mM | Use of NPs significantly increased the wheat growth, biomass production, chlorophyll contents (a, b, and carotenoids), reduction in lipid peroxidation and electrolyte leakage along with timey production of antioxidants (SOD, POD, APX) against the ROS (H_2_O_2_, (O_2_^•-, •^OH) under DS. | ([Noor et al., 2022](#_ENREF_119)) |

**Table S3: Effect of nano-particles (NPs) on oxidative stress markers, antioxidant activities and gene expression under drought stress**

| **Crop** | **Drought stress** | **NPs type** | **NPs concentration** | **Effects** | **Reference** |
| --- | --- | --- | --- | --- | --- |
| Feverfew | 120 days with different irrigation gap of 4, 8 and 12 days | Glycine-nano-silicon combination | 3.0mM | Nano particle complex reduced membrane damage index (MDI), H_2_O_2_, MDA and phenol with increasing antioxidants in severe drought conditions | ([Esmaili et al., 2022](#_ENREF_48)) |
| Green pea | 10 days | SiO2 NPs | 50ppm | NPs increased tolerance by increasing the activity of antioxidant enzymes (SOD, CAT, GR) and reduce lipid peroxidation and H_2_O_2_ concentration in plant tissues. Additionally, phenolic compounds and non-enzymatic antioxidant activities (DPPH, ABTS, FRAP) increased under DS. | ([Sutulienė et al., 2021](#_ENREF_164)) |
| Cape periwinkle | 50% FC | Chitosan nanoparticles (CSNPs) | 1% | CSNPs increased the activities of CAT and APX, induced the antioxidant potential and gene expression of alkaloid biosynthesis while reducing the H_2_O_2_ and MDA accumulation to protect plant from lipid peroxidation under DS. | ([Ali et al., 2021a](#_ENREF_16)) |
| Sorghum | 21 days | CeO_2_NPs | (10 mg L^–1^) | NPs decreased cell membrane lipid peroxidation and reduced hydrogen peroxide levels along with leaf superoxide radical under DS. | ([Djanaguiraman et al., 2018](#_ENREF_40)) |
| Barley | 50% and 25% FC | Nano-SiO_2_ | 125 and 250 mg L^−1^ | The antioxidant enzymes activities, membrane stability indices, osmolyte concentrations and carotenoid level were significantly increased with nano-SiO_2_ application under DS. | ([Ghorbanpour et al., 2020](#_ENREF_61)) |
| Wheat | 40% FC | SiO_2_ NPs | 150mg/L | NPs significantly increased sugar and proline contents with increasing antioxidant (SOD, POD, and CAT) enzymes. | ([Akhtar et al., 2021](#_ENREF_9)) |
| Lemon balm | 40% FC | Fe-NPs | 20 μM | Fe NPs also resulted in alleviating oxidative stress by reducing total antioxidant activity (TAA), total phenolic content (TPC) and increasing antioxidant enzymes (proline) accumulation under DS. | ([Mohasseli et al., 2020](#_ENREF_114)) |

**Table S4: Effect of nano-particles (NPs) types, characterization and potential mechanism of NPs in inducing the drought stress**

| **Crop species** | **Nano-Particle Concentration** | **Nano-particles characterization** | **Potential mechanism to induced drought stress** | **Reference** |
| --- | --- | --- | --- | --- |
| *Salvia abrotanoides* | Chitosan NPs (60 and 90 ppm) | CNPs were produced by chemical method and they had 50 nm size with purity of 99%. | CNPs reduced the negative effects of DS by increasing RWC, chlorophyll, carotenoid, phenols, flavonoid, soluble sugar and proline concentration and antioxidant activity. | (Attaran et al., 2022) |
| Sunflower | TiO_2_-NPs (300 and 600 ppm) | TiO_2_-NPs were prepared by chemical method and they have 99.70% purity along with 25 nm size. | TiO_2_-NPs mitigated the adverse effects of DS by decreasing lipid per-oxidation, H_2_O_2_ production and increasing the CAT and GPX activity, and proline accumulation. | (Ramdan et al., 2022) |
| Wheat | ZnO-NPs (100 mg L^-1^) | ZnO-NPs were produced by biological method and they have surface area of 12.35 m^2^·g^−1^. | ZnO-NPs mitigated the adverse effects of DS by increasing proline synthesis, antioxidant activities and chlorophyll contents. | (Abd-El-Aziz et al., 2022). |
| Maize | Cu-NPs (4.44 and 5.55 mg L^-1^) | Cu-NPs were prepared by chemical method and they have a size of 30-40 nm. | Cu-NPs reduced the negative effects of DS by increasing chlorophyll, anthocyanin, carotenoid contents, antioxidant activities and decreasing ROS production. | (Van-Nguyen et al., 2022) |
| Rice | ZnO-NPs (25 and 50 ppm) | ZnO-NPs were produced by chemical method and have 98% purity, 20-30 nm size. | ZnO-NPs improved the rice yield by increasing chlorophyll synthesis and activity of CAT, POD and SOD. | (Waqas et al., 2022) |
| Coriander | Si-NPs (1.5 mM) | Si-NPs were produced by chemical method and they have 99% purity and size of 25-35 nm. | The application of NPs increased yield mitigated the adverse effects of DS by increasing RWC, soluble sugars, total phenolic and flavonoid contents. | (Afshari et al., 2021) |
| Rice | Hg-NPs (50 and 100 mg kg^-1^) | Hg-NPs were synthesized chemically and they have size of 18-94 nm. | Hg based NPs improved the growth and reduced the adverse effects of DS by decreasing MDA and ROS production owing to increase in antioxidant (CAT, POD and SOD) activity, proline accumulation, and nutrient uptake and genes expression. | (Ahmed et al., 2021) |
| Wheat | TiO_2_-NPs (20 and 40 ppm) | TiO_2_-NPs were prepared by biological method. | TiO2-NPs improved growth by increasing chlorophyll and sugar contents, membrane stability, antioxidant activity and uptake of NPK. | (Mustafa et al., 2021) |
| Strawberry | SiO_2_-NPs (50 and 100 mg L^-1^) | SiO_2_-NPs were produced by chemical method and they have 99% purity. | SiO_2_-NPs improved the growth under DS by increasing RWC, membrane stability, water use efficiency, antioxidant activities, total phenolic and anthocyanin contents. | (Zahedi et al., 2020b) |
| Wheat | TiO_2_-NPs (1000 and 2000 mg L^-1^) | TiO_2_-NPs were prepared by chemical method and they have 99% purity, along with 10-25 nm size and bulk density of 0.24 g cm^-3^. | The application of TiO_2_-NPs reduced adverse effects of DS by increasing germination, root and shoot growth and vigor index. | (Faraji et al., 2019) |

**References**

Bahadur, A., Chatterjee, A., Kumar, R., Singh, M., and Naik, P. (2011). Physiological and biochemical basis of drought tolerance in vegetables. *Veg. Sci*. 38, 1–16.

Jafarnia, S., Akbarinia, M., Hosseinpour, B., Modarres Sanavi, S., and Salami, S. A. (2018). Effect of drought stress on some growth, morphological, physiological, and biochemical parameters of two different populations of *Quercus brantii*. *Forest-Biogeosci. Forestry*. 11, 212. doi: 10.3832/ifor2496-010

Batool, M., El-Badri, A. M., Wang, Z., Mohamed, I. A., Yang, H., Ai, X., et al. (2022). Rapeseed morpho-physio-biochemical responses to drought stress induced by PEG-6000. *Agronomy* 12, 579. doi: 10.3390/agronomy12030579

Noein, B., and Soleymani, A. (2022). Corn (*Zea mays* l.) physiology and yield affected by plant growth regulators under drought stress. *J. Plant Growth Regul*. 41, 672–681. doi: 10.1007/s00344-021-10332-3

Mannan, M., Tithi, M. A., Islam, M. R., Al Mamun, M., Mia, S., Rahman, M., et al. (2022). Soil and foliar applications of zinc sulfate and iron sulfate alleviate the destructive impacts of drought stress in wheat. *Cer. Res. Comm*., 1–11. doi: 10.1007/s42976-022-00262-5

Gurumurthy, S., Sarkar, B., Vanaja, M., Lakshmi, J., Yadav, S., and Maheswari, M. (2019). Morpho-physiological and biochemical changes in black gram (*Vigna mungo* l. hepper) genotypes under drought stress at flowering stage. *Acta Physiol. Plant* 41, 1–14. doi: 10.1007/s11738-019-2833-x

Akhtar, N., Ilyas, N., Meraj, T. A., Pour-Aboughadareh, A., Sayyed, R., Mashwani, Z. U. R., et al. (2022). Improvement of plant responses by nanobiofertilizer: A step towards sustainable agriculture. *Nanomaterials* 12, 965. doi: 10.3390/nano12060965

Hussein, H.-a. A., Alshammari, S. O., Kenawy, S. K., Elkady, F. M., and Badawy, A. A. (2022). Grain-priming with l-arginine improves the growth performance of wheat (*Triticum aestivum* l.) plants under drought stress. *Plants* 11, 1219. doi: 10.3390/plants11091219

Ahmad, S., Wang, G. Y., Muhammad, I., Chi, Y. X., Zeeshan, M., Nasar, J., et al. (2022). Interactive effects of melatonin and nitrogen improve drought tolerance of maize seedlings by regulating growth and physiochemical attributes. *Antioxidants* 11, 359. doi: 10.3390/antiox11020359

Waqas Mazhar, M., Ishtiaq, M., Hussain, I., Parveen, A., Hayat Bhatti, K., Azeem, M., et al. (2022). Seed nano-priming with zinc oxide nanoparticles in rice mitigates drought and enhances agronomic profile. *PloS One* 17, e0264967. doi: 10.1371/journal.pone.0264967

Ghani, M. I., Saleem, S., Rather, S. A., Rehmani, M. S., Alamri, S., Rajput, V. D., et al. (2022). Foliar application of zinc oxide nanoparticles: An effective strategy to mitigate drought stress in cucumber seedling by modulating antioxidant defense system and osmolytes accumulation. *Chemosphere* 289, 133202. doi: 10.1016/j.chemosphere.2021.133202

Van Nguyen, D., Nguyen, H. M., Le, N. T., Nguyen, K. H., Nguyen, H. T., Le, H. M., et al. (2022). Copper nanoparticle application enhances plant growth and grain yield in maize under drought stress conditions. *J. Plant Growth Reg*. 41, 364–375. doi: 10.1007/s00344-021-10301-w

Azmat, A., Tanveer, Y., Yasmin, H., Hassan, M. N., Shahzad, A., Reddy, M., et al. (2022). Coactive role of zinc oxide nanoparticles and plant growth promoting rhizobacteria for mitigation of synchronized effects of heat and drought stress in wheat plants. *Chemosphere* 297, 133982. doi: 10.1016/j.chemosphere.2022.133982

Ikram, M., Raja, N. I., Javed, B., Hussain, M., Hussain, M., Ehsan, M., et al. (2020). Foliar applications of bio-fabricated selenium nanoparticles to improve the growth of wheat plants under drought stress. *Green Proc. Syn*. 9, 706–714. doi:10.1515/gps-2020-0067

Rahimi, S., Hatami, M., and Ghorbanpour, M. (2021). Silicon-nanoparticle mediated changes in seed germination and vigor index of marigold (*Calendula officinalis* l.) compared to silicate under PEG-induced drought stress. *Ges. Pfla*. 73, 575–589. doi: 10.1007/s10343-021-00579-x

Noor, R., Yasmin, H., Ilyas, N., Nosheen, A., Hassan, M. N., Mumtaz, S., et al. (2022). Comparative analysis of iron oxide nanoparticles synthesized from ginger (*Zingiber officinale*) and cumin seeds (*Cuminum cyminum*) to induce resistance in wheat against drought stress. *Chemosphere* 292, 133201. doi: 10.1016/j.chemosphere.2021.133201

Esmaili, S., Tavallali, V., Amiri, B., Bazrafshan, F., and Sharafzadeh, S. (2022). Foliar application of nano-silicon complexes on growth, oxidative damage and bioactive compounds of feverfew under drought stress. *Silicon* 1–12. doi: 10.1007/s12633-022-01754-z

Sutulienė, R., Ragelienė, L., Samuolienė, G., Brazaitytė, A., Urbutis, M., and Miliauskienė, J. (2021). The response of antioxidant system of drought-stressed green pea (*Pisum sativum* l.) affected by watering and foliar spray with silica nanoparticles. *Horticulturae* 8, 35. doi: 10.3390/horticulturae8010035

Ali, E., El-Shehawi, A., Ibrahim, O., Abdul-Hafeez, E., Moussa, M., and Hassan, F. (2021a). A vital role of chitosan nanoparticles in improvisation the drought stress tolerance in *Catharanthus roseus* (L.) through biochemical and gene expression modulation. *Plant Physiol. Biochem*. 161, 166–175. doi: 10.1016/j.plaphy.2021.02.008

Djanaguiraman, M., Nair, R., Giraldo, J. P., and Prasad, P. V. V. (2018). Cerium oxide nanoparticles decrease drought-induced oxidative damage in sorghum leading to higher photosynthesis and grain yield. *ACS Omega*. 3, 14406–14416. doi: 10.1021/acsomega.8b01894

Ghorbanpour, M., Mohammadi, H., and Kariman, K. (2020). Nanosilicon-based recovery of barley (*Hordeum vulgare*) plants subjected to drought stress. *Environ. Sci.: Nano*. 7, 443–461. doi: 10.1039/C9EN00973F

Akhtar, N., Ilyas, N., Hayat, R., Yasmin, H., Noureldeen, A., and Ahmad, P. (2021). Synergistic effects of plant growth promoting rhizobacteria and silicon dioxide nano-particles for amelioration of drought stress in wheat. *Plant Physiol. Biochem*. 166, 160–176. doi: 10.1016/j.plaphy.2021.05.039

Mohasseli, V., Farbood, F., and Moradi, A. (2020). Antioxidant defense and metabolic responses of lemon balm (*Melissa officinalis* l.) to Fe-nano-particles under reduced irrigation regimes. *Indust. Crops Prod*. 149, 112338. doi: 10.1016/j.indcrop.2020.112338

Attaran, D. S., Karimian, Z., Mostafaei Dehnavi, M., and Samiei, L. (2022). Chitosan nanoparticles improve physiological and biochemical responses of *Salvia abrotanoides* (Kar.) under drought stress. *BMC Plant Biol*. 22 (1), pp.1–pp17. doi: 10.1186/s12870-022-03689-4

Ramadan, T., Sayed, S. A., Abd-Elaal, A. K., and Amro, A. (2022). The combined effect of water deficit stress and TiO_2_ nanoparticles on cell membrane and antioxidant enzymes in *Helianthus annuus* l. Physiol. *Mol. Biol. Plant* 28 (2), pp.391–pp.409. doi: 10.1007/s12298-022-01153-z

Abd-El-Aziz, G. H., Ahmed, S. S., Radwan, K. H., and Fahmy, A. H. (2022). Positive and negative environmental effect of using zinc oxide nanoparticles on wheat under drought stress. *Open J. App. Sci*. 12 (6), 1026–1044. doi: 10.4236/ojapps.2022.126070

Waqas Mazhar, M., Ishtiaq, M., Hussain, I., Parveen, A., Hayat Bhatti, K., Azeem, M., et al. (2022). Seed nano-priming with zinc oxide nanoparticles in rice mitigates drought and enhances agronomic profile. *PloS One* 17, e0264967.doi: 10.1371/journal.pone.0264967

Afshari, M., Pazoki, A., and Sadeghipour, O. (2021). Foliar-applied silicon and its nanoparticles stimulate physio-chemical changes to improve growth, yield and active constituents of coriander (*Coriandrum sativum* l.) essential oil under different irrigation regimes. *Silicon* 13 (11), pp.4177–4188. doi: 10.1007/s12633-021-01101-8

Ahmed, T., Noman, M., Manzoor, N., Shahid, M., Abdullah, M., Ali, L., et al. (2021). Nanoparticle-based amelioration of drought stress and cadmium toxicity in rice via triggering the stress responsive genetic mechanisms and nutrient acquisition. *Ecotoxicol. Environ. Saf*. 209, 111829. doi: 10.1016/j.ecoenv.2020.111829

Mustafa, H., Ilyas, N., Akhtar, N., Raja, N. I., Zainab, T., Shah, T., et al. (2021). Biosynthesis and characterization of titanium dioxide nanoparticles and its effects along with calcium phosphate on physicochemical attributes of wheat under drought stress. *Ecotoxicol. Environ. Saf*. 223, 112519. doi: 10.1016/j.ecoenv.2021.112519

Zahedi, S. M., Moharrami, F., Sarikhani, S., and Padervand, M. (2020b). Selenium and silica nanostructure-based recovery of strawberry plants subjected to drought stress. *Sci. Rep*. 10, 1–18. doi: 10.1038/s41598-020-74273-9

Faraji, J., and Sepehri, A. (2019). Ameliorative effects of TiO_2_ nanoparticles and sodium nitroprusside on seed germination and seedling growth of wheat under PEG-stimulated drought stress. *J. Seed. Sci*. 41, 309–317. doi: 10.1590/2317-1545v41n3213139
